# Effectiveness of Multidrug Antiretroviral Regimens to Prevent Mother-to-Child Transmission of HIV-1 in Routine Public Health Services in Cameroon

**DOI:** 10.1371/journal.pone.0010411

**Published:** 2010-04-29

**Authors:** Patrice Tchendjou, Chantal Same-Ekobo, Annie Nga, Mathurin Tejiokem, Anfumbom Kfutwah, Anne Njom Nlend, Landry Tsague, Anne Cécile Bissek, Daniel Ekoa, Joanna Orne-Gliemann, Dominique Rousset, Régis Pouillot, François Dabis

**Affiliations:** 1 Laboratoire Epidémiologie, Centre Pasteur du Cameroun, Yaoundé, Cameroon; 2 Centre Hospitalier Essos, Yaoundé, Cameroon; 3 Laboratoire de Virologie, Centre Pasteur Cameroun, Yaoundé, Cameroon; 4 Direction de la Lutte contre la Maladie, Ministère de la Santé Publique du Cameroun, Yaoundé, Cameroon; 5 INSERM U 897, Institut de Santé Publique Epidémiologie et Développement, Bordeaux, France; University of Cape Town, South Africa

## Abstract

**Background:**

Multidrug antiretroviral (ARV) regimens including HAART and short-course dual antiretroviral (sc-dARV) regimens were introduced in 2004 to improve Prevention of Mother-to-Child Transmission (PMTCT) in Cameroon. We assessed the effectiveness of these regimens from 6–10 weeks and 12 months of age, respectively.

**Methodology/Findings:**

We conducted a retrospective cohort study covering the period from October 2004 to March 2008 in a reference hospital in Cameroon. HIV-positive pregnant women with CD4 ≤350 cells/mm^3^ received first-line HAART [regimen 1] while the others received ARV prophylaxis including sc-dARV or single dose nevirapine (sd-NVP). Sc-dARV included at least two drugs according to different gestational ages: zidovudine (ZDV) from 28–32 weeks plus sd-NVP [regimen 2], ZDV and lamuvidine (3TC) from 33–36 weeks plus sd-NVP [regimen 3]. When gestational age was ≥37 weeks, women received sd-NVP during labour [regimen 4]. Infants received sd-NVP plus ZDV and 3TC for 7 days or 30 days. Early diagnosis (6–10 weeks) was done, using b-DNA and subsequently RT-PCR. We determined early MTCT rate and associated risk factors using logistic regression. The 12-month HIV-free survival was assessed using Cox regression. Among 418 mothers, 335 (80%) received multidrug ARV regimens (1, 2, and 3) and MTCT rate with multidrug regimens was 6.6% [95%CI: 4.3–9.6] at 6 weeks, without any significant difference between regimens. Duration of mother's ARV regimen <4 weeks [OR = 4.7, 95%CI: 1.3–17.6], mother's CD4 <350 cells/mm^3^ [OR = 6.4, 95%CI: 1.8–22.5] and low birth weight [OR = 4.0, 95%CI: 1.4–11.3] were associated with early MTCT. By 12 months, mixed feeding [HR = 8.7, 95%CI: 3.6–20.6], prematurity [HR = 2.3, 95%CI: 1.2–4.3] and low birth weight were associated with children's risk of progressing to infection or death.

**Conclusions:**

Multidrug ARV regimens for PMTCT are feasible and effective in routine reference hospital. Early initiation of ARV during pregnancy and proper obstetrical care are essential to improve PMTCT.

## Introduction

Infection due to Human Immunodeficiency Virus type 1 (HIV-1) is one of the main causes of morbidity and mortality among children in Africa [1], [2]. By the end of 2007, 33.2 million people were living with HIV/AIDS in the world, of whom about 2.5 million were children under 15 years [3]. More than 95% of all HIV-infected children are living in Africa, and most of them have acquired HIV through Mother-To-Child Transmission (MTCT) [4], [5]. In the absence of interventions to prevent MTCT of HIV-1, transmission rates can reach 40% in breastfeeding populations [6]. Global estimations in 2007 show that 1,500 children were infected everyday by HIV-1 and that 330,000 deaths in children were due to AIDS-related conditions [3].

However, in most cases, a number of preventive measures are available to drastically reduce cases of MTCT of HIV-1. In most developed countries and some middle-income countries, such as Brazil and Thailand, implementation of effective strategies including Highly Active Antiretroviral Therapy (HAART) with scheduled caesarean sections and avoidance of breastfeeding have reduced MTCT rate to below 2% [3], [4]. In resource-constrained settings, elective caesarean delivery is seldom feasible [7] and it is often neither acceptable nor safe for mothers to refrain from breastfeeding. In these settings, initial efforts to prevent HIV infection in infants were focused on reducing MTCT around the period of labour and delivery, which accounts for one to two thirds of the overall transmission, depending on whether the mother breastfeeds or not. Antiretroviral (ARV) prophylaxis around the time of delivery alone can reduce the risk of MTCT in a breastfeeding population to almost two-fold following vaginal delivery (41–47% reduction in risk) [8], [9]. Further studies in sub-Saharan Africa demonstrated that multidrug ARV regimens had greater efficacy than sd-NVP [2], [10]. MTCT rates as low as 5.6% was reported with short-course (sc) multidrug ARV regimens combined with shortened breastfeeding in a research setting in Abidjan [11].

In 2004, WHO revised recommendations for the use of antiretroviral drugs for treating pregnant women and preventing HIV infection in infants, stipulating that pregnant women who need HAART for their own health should receive first-line ARV regimens as soon as possible during pregnancy. HIV-infected pregnant women without indications for antiretroviral treatment should mainly receive ARV regimen including zidovudine (ZDV) starting from 28 weeks of pregnancy plus sd-NVP and ZDV during labour, and sd-NVP plus ZDV for one week should be given to the infant [12]. Programmatic considerations and health systems constraints in most high-burden countries should be taken into consideration while adapting ARV guidelines for national PMTCT programmes. However, little information on field effectiveness of the 2004 WHO guidelines is available in the literature.

With a prevalence of HIV among pregnant women estimated at 7.3% in 2007 [3], about 20,000 new HIV infections in infants are expected each year in Cameroon without any intervention. A pilot PMTCT initiative supported by the Glaxo Smith Kline Foundation started in year 2000 and reported a MTCT rate of 13.1% at 24 months using the HIVNET-012 sd-NVP regimen [13]. The national PMTCT program was launched in 2002 based on the early results of this pilot initiative, and maternal and infant sd-NVP was recommended as first choice PMTCT regimen. In 2004, following preliminary results from the “ANRS 1201/1202 trial” reported in 2002 and according to revised WHO guidelines, the Cameroonian Ministry of Health revised the national guidelines for PMTCT and recommended treatment with HAART for HIV-positive pregnant women with CD4 counts ≤350 cells/mm3 and short-course combination regimens for pregnant women without indication of treatment. In 2007, a demonstrative program carried out in Abidjan reported a safe and highly effectiveness of HAART and sc ARV regimens [14]. However, data were still lacking on the routine effectiveness of these new regimens in a resource-constrained setting and should ideally be reported as early as possible. The hypothesis assessed in this study was that the implementation of the revised multidrug ARV regimens for PMTCT would result in routine in transmission rates decreasing from 13.1% (as earlier reported in Yaounde with sd-NVP regimen [13]) to a value which could be close to 6% (the value reported in Abidjan between 2001 and 2005 [15]). We report a 6–10 weeks and 12 months effectiveness of the new regimens in a day-to-day practice in a reference hospital in Yaounde, Cameroon.

## Methods

### Study site

The study was conducted at the “Caisse Nationale de Prevoyance Sociale (CNPS)” hospital, a reference semi-private health facility in Yaounde, Cameroon, where PMTCT was implemented since 2000. The prevalence of HIV infection among pregnant women in Yaounde was estimated around 8% in 2004 [16]. Pregnant women received at the CNPS were characterised by their interest and willingness to use existing PMTCT services in this reference hospital. These included all pregnant women who consulted during the study period and were diagnosed HIV positive during ANC visits, and known HIV-positive women who became pregnant and attended ANC visits at this hospital during the consider time. Children involved in the study were those born to the above mentioned groups of women. In this structure, the PMTCT package proposed include the following: Voluntary counselling and testing “opt out”, clinical and immunological assessment of women identified as HIV-infected, HAART or ARV prophylaxis, safer delivery practices, counselling and care related to nutrition (exclusive formula feeding or shortened breastfeeding) and psychosocial support, early HIV-diagnosis of children and follow-up of exposed children up to 12 months.

### Study design

We conducted a retrospective cohort study nested within a routine PMTCT program implemented according to national guidelines. The period covered by this study was October 2004 to March 2008.

### Study population

HIV-positive pregnant women aged 18 years and older, who had received ARV (sd-NVP, sc-dARV and HAART) at the CNPS hospital, were eligible for the study with their neonates. These children had received ARV drugs at birth, and had been followed-up between birth and 12 months of age. None of the participating women were involved in any clinical trial during the study period. Exclusion criteria from the study included being a second twin or a third triplet since studies have reported a lower MTCT risk for second twins and third triplets [17].

To assess the effectiveness of the new recommendations, it is important for the study to have a sufficient statistical power to demonstrate that there is a significant difference between the expected transmission rate and the transmission rate observed earlier. The minimal sample size for this comparison between expected MTCT rate (which can go down to 6%) and the observed MTCT rate (13.1%) is 223 mother-child pairs. Since files were accessible, we included more participating women who met the eligibility criteria than the minimal size required.

### Antiretroviral interventions to prevent MTCT of HIV

CD4 cell counts of enrolled patients were performed using the flow cytometry technique (Becton Dickinson FACSCount™ System). From October 2004 to August 2006, HIV-positive pregnant women with CD4 counts inferior to 200 cells/mm^3^ were eligible to receive HAART (according to current national guidelines) referred in this report as regimen 1. The regimen 1 included ZDV+3TC+NVP or D4T+3TC+NVP. HIV-positive pregnant women with CD4 count ≥200 cells/mm^3^ received short-course dual antiretroviral (sc-dARV) regimens, comprising short-course zidovudine (sc ZDV) in antepartum and sd-NVP during labour, or short-course of zidovudine+lamivudine (sc ZDV+3TC) in antepartum and sd-NVP during labour. Some women of the latter group did not receive ARV in antepartum. These ARV regimens were initiated depending on the gestational ages of pregnancies calculated from the last menstrual period: 28–32 weeks [regimen 2; sc ZDV in antepartum+sd-NVP and ZDV/3TC during labour+7 days ZDV/3TC in post partum]; 33–36 weeks [regimen 3; sc (ZDV/3TC) in antepartum+sd-NVP during labour+7 days ZDV/3TC in post partum] and 37 weeks or older [regimen 4; sd-NVP during labour+7 days ZDV/3TC in post partum]. Infants received sd-NVP+7 days ZDV (or ZDV+3TC) as standard complementary ARV regimen, or 30 days ZDV (or ZDV+3TC) if the mother had not received ARV during antenatal period. Between September 2006 and March 2008, the CD4 count threshold for HAART eligibility was raised to 350cells/mm^3^, but the ARV regimens remained unchanged. All regimens were administered orally. In addition, all women received individual counselling on feeding practices as recommended by WHO. Women who chosed replacement feeding were educated on safe formula preparation and administration. Psychosocial support was offered. Apart from scheduled visits, no specific strategy to document compliance was developed.

### Follow up of participants

PMTCT interventions were administered at the antenatal clinic, laboratory, maternity and paediatric services. These services involved physicians, counsellors (voluntary HIV testing and nutrition), mid-wives, psychologists and social workers. Pregnant women were seen every month if the gestational age was 28–36 weeks, and every two weeks beyond 36 weeks. All women were encouraged to deliver in health facilities.

At birth, all children were examined by a paediatrician before discharged from the hospital. During the first visit between 6–10 weeks, in addition to clinical, psychological and nutritional follow-up, early diagnosis of HIV-1 infection was proposed. Before 2007, HIV diagnosis was performed by quantification of viral RNA in plasma samples using the branched DNA (b-DNA) technique (Bayer Diagnostics, Paris, France). In 2007, a real time PCR (RT-PCR) using the TaqMan technology, an in-house protocol validated by the French “Agence Nationale de Recherche sur le Sida et les hépatites virales” (ANRS, AC 11 working group, France) replaced the b-DNA technique. Diagnosis was performed by the virology laboratory of the Centre Pasteur du Cameroun. Positive results were confirmed with a second infant specimen. All infants who were diagnosed with an early HIV-1 infection and who came back to the clinic were enrolled into the care and treatment program and initiated the antiretroviral therapy. After the first visit (6–10 weeks), children were followed every month until the age of 4 months and every trimester through the age of 15 months. At each visit, clinical and nutritional assessments were performed. Mothers were strongly encouraged to bring their children to hospital if there was any medical incident. For children aged from 9 to 12 months, peripheral blood was obtained at least three months after cessation of breastfeeding (when this was the feeding option) and tested for HIV using ELISA (enzyme-linked immunosorbent assay) technique. All confirmed HIV-positive children who returned for results were systematically enrolled into the care and treatment program and initiated on HAART.

### Study end points

The primary end point was HIV infection through the scheduled 6-week visit window (diagnosis made between 6 and 10 weeks). By 12 months of age, children were either HIV infected, uninfected, lost to follow-up or dead. The composite end point was HIV-free survival through to 12 months with a visit window which could go up to two weeks.

### Data collection

At each scheduled visit, information on infant's feeding practice, morbidity; mortality and all clinical events up till the age of one year were documented using patient files or registers. A questionnaire was designed to collect mother-infant pair's variables. Information missing in the medical files was abstracted from service registers. Feeding practice variables were defined according to the following: children whose mothers reported exclusive breastfeeding at each scheduled visit between birth and 6 months were considered as exclusively breastfed; children whose mothers reported exclusive formula feeding at each scheduled visit between birth and 6 months were considered as exclusively formula-fed; and children whose mothers reported simultaneous breastfeeding and formula feeding or interrupted periods of breastfeeding with formula feeding in an inconsistent manner were considered as mixed-fed. All recorded variables were classified as either dependent or independent. Dependent variables were child HIV status at six week after birth (infected versus uninfected) and HIV-free survival of children at 12-month (free of HIV and alive versus infected or dead). Mother-related independent variables were age, educational level, marital status, number of pregnancy, parity, premature rupture of membrane defined as rupture occurring before the beginning of the labour whatever the moment, mode of delivery, term of pregnancy (children born before 37 weeks of pregnancy were considered premature), ARV regimen and its duration, and CD4 cell counts. Children-related independent variables were date of birth, birth weight, gender, feeding practice at each scheduled visit between birth and six months, type of ARV regimen and clinical events between scheduled visits.

### Statistical methods

Mothers' treatment had 4 modalities: regimen 1, 2, 3 and 4. Mothers were divided into 2 categories according to CD4 counts during pregnancy: CD4 <200 cells/mm^3^ versus CD4 ≥200cells/mm^3^ between October 2004 and August 2006; CD4 <350 cells/mm^3^ versus CD4 ≥350cells/mm^3^ from September 2006 to March 2008. We assumed that feeding practices reported at each visit was the one effectively done. Bivariate analyses were done using the Student's t-test to compare continuous variables and the Chi-square test to compare categorical variables. A p value<0.05 was considered significant.

Early HIV infection was used to assess short-term efficacy (6–10weeks), while HIV-free survival were used to assess long-term efficacy (12 months) of interventions implemented in routine, based on international recommendations [18]. Determinants of infant probability to become infected at 6-week was estimated using a multivariate logistic regression model [19]. We defined postnatal transmission as a child with negative HIV PCR from a sample obtained between 6–10 weeks and who later became infected. Time to event was defined as the delay between birth and the occurrence of an event (infection or death) or between birth and reaching end point. All variables associated to early child infection in univariate analysis with a significance level<0.20 were included in the stepwise multivariate analyses, with consideration for confounding effect and interactions. The final multivariate model contained the variables with a significance level<0.05, but some other variables (such as mode of delivery) could be forced into the model when being considered of particular interest according to previous studies [11].

For HIV-free survival analysis, the time of the event occurrence was the date of death or the date when HIV infection was diagnosed. Data from infants lost to follow-up before reaching 12-month were censored. Probabilities of 12-month HIV-free survival for children exclusively breastfed, children exclusively formula-fed or children who practiced mixed feeding were estimated using the Kaplan-Meier methods with *P* values calculated using the log-rank test. Cox proportional hazards regression models [20] were used to explore determinants of HIV-free survival at 12-month. The choice of variables to be included in the final multivariate Cox model was determined using a backward stepwise procedure. All variables that met the proportional hazard assumption and associated, in univariate analysis, with the event with a significance level<0.20 were first included in the model. The final multivariate model contained the variables with a significance level<0.05. Data were managed using Epidata 3.1 software and analyzed using SAS 9.1 software (SAS Institute Inc., Cary, NC, USA). Analyses were made on an intention-to-treat basis.

### Ethical and administrative aspects

PMTCT interventions at CNPS hospital were implemented according to the national guidelines of Cameroon, under the supervision of the Ministry of Public Health (MOH). This retrospective evaluation of MOH's recommendations was approved by the technical medical division (TMD) of the CNPS hospital, the board in charge of the ethics of practices and research activities in the CNPS hospital, which gave the authorization.

## Results

### General characteristics

Between October 2004 and March 2008, 443 mother-infant pairs were received at the site. Among these pairs, 25 (5.6%) were considered as unexploitable data because either the children were lost to follow-up before 6 weeks of age or the files of the mothers and/or infants were incomplete. 418 eligible mothers with their children were retained for analysis. The median age of mothers was 27 years (interquartile range [IQR] 24–30 years). More than 74% (310/418) of them had at least secondary school level education, 68% were experiencing their first pregnancy or had only one previous delivery. Of 418 enrolled women, 73 (17.5%) had received regimen 1 at a median gestational age of 32 weeks, 163 (38.9%) had received regimen 2, 99 (23.7%) had received regimen 3 and 83 (19.9%) had received regimen 4. During pregnancy, CD4 cell counts were done in the weeks that followed the diagnosis of HIV or following the first ANC visit for known HIV-positive women. The median CD4 count was 380cells/mm^3^ (IQR 310–450cells/mm^3^). Thirty-six women (8.6%) delivered through caesarean section. Among the 418 live births, 11.2% were born to mothers who had a premature rupture of membranes. The median weight at birth was 3,000 grams (IQR 2550–3400 grams). Overall, 22.2% (93/418) had a low birth weight (<2,500 grams) and 16.3% (68/418) were born before 37 weeks of amenorrhea. More than 85% (357/418) infants were classified as exclusively formula-fed (EFF), 9.1% (38/418) were classified as exclusively breastfed (EBF) and 5.5% (23/418) were classified as mixed-fed (MF).

### MTCT rates

**Figure 1:**
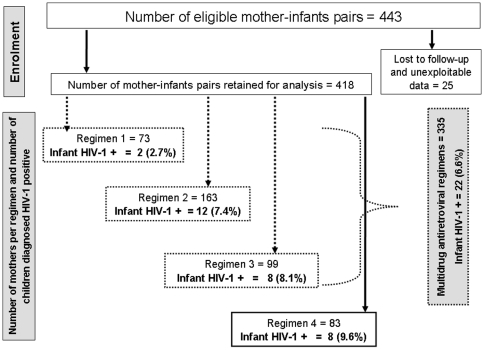
Flowchart describing regimens received and MTCT rates at six weeks at the CNPS, Cameroon. Between October 2004 and March 2008, 443 mother-infant pairs were eligible for the study. Among these, 25 had unexploitable data and 418 were retained for analysis.

Early (6–10 weeks) MTCT rate of 2.7% [2/73; 95% CI: 0.5–8.8], 7.4% [12/163; 95% CI: 4.1–12.2], 8.1% [8/99; 95% CI: 3.8–14.8] and 9.6% [8/83; 95% CI: 4.6–17.5] were registered respectively with regimens 1, 2, 3 and 4 (figure 1). Taking in consideration only women having received multidrug ARV regimens prior delivery (1, 2 and 3), overall MTCT rate was 6.6% [22/335; 95%CI: 4.3–9.6] at 6–10 weeks. At 12-months, late cumulative MTCT rate regardless of regimen type (1 to 4) was 8.1% [32/394; 95%CI: 5.7–11.1]. Postnatal transmission at 12 months was 25% [8/32; 95%IC: 12.4–42] of infected children. This occurred throughout breastfeeding. Taking into account the changes in HAART eligibility in the course of the study (October 2004 to August 2006; September 2006 to March 2008), we did not find any difference in MTCT rate after the use of multidrug regimens (8.1% Vs 4.6%; p = 0.18).

### Factors associated with 6–10 weeks MTCT of HIV

In univariate analysis, there was no significant difference in early MTCT rate between the ARV regimens. Mothers with CD4 counts below 350cells/mm^3^ had a fourfold risk (OR: 4.0; 95%CI: 1.7–9.2) of MTCT. The following factors were found to be associated to a high risk of early MTCT: a duration of treatment shorter than 4 weeks (OR: 6.6; 95%CI: 2.9–15.3), premature rupture of membranes (OR: 39.9; 95%CI: 16.2–98.7), low birth weight (OR: 8.6; 95%CI: 3.9–19.2) and prematurity (OR: 3.9; 95%CI: 1.8–8.7). Except for prematurity, the other variables associated with early MTCT risk in univariate analysis were still found to be associated with high MTCT risk in multivariate analysis (table 1).

**Table 1:** Determinants of the 6-weeks MTCT Risk (Multivariate logistic regression model), at the CNPS hospital of Yaounde in Cameroon between 2004 and 2008.

	N	OR**	95% CI	P	ORa***	95% CI	*P*
**Duration of treatment**							
≥4 weeks before delivery	306	1			1		
<4 weeks before delivery	112	6.6	2.9–15.3	**<0.0001**	4.7	1.3–17.6	**<0.0001**
**Treatment**							
Regimen 1 (HAART)	73	1			1		
Regimen 2	163	2.8	0.6–12.9	0.18	0.5	0.05–4.9	0.5
Regimen 3	99	3.1	0.6–15.2	0.15	1.6	0.2–12	0.6
Regimen 4	83	3.7	0.8–18.4	0.15	2.1	0.2–18.5	0.5
**TCD4 Lymphocytes**							
>350 cells/ml	238	1			1		
≤350 cells/ml	180	4.02	1.7–9.2	**<0.001**	6.4	1.8–22.5	**<0,004**
**Delivery**							
Cesarean section	36	1			1		
Normal	382	2.9	0.4–21.7	0.30	2.4	0.2–25.9	0.46
**Premature rupture of membranes**							
No	371	1			1		
Yes (before onset of labour)	47	39.9	16.2–98.7	**<0.0001**	27.6	8.9–84.8	**0,0001**
**Birth weight**							
>2500 grams	325	1			1		
≤2500 grams	93	8.6	3.9–19.2	**0.0001**	4.0	1.4–11.3	**0,008**
**Prematurity** *							
No	68	1			1		
Yes	350	3.9	1.8–8.7	0.01	0.82	0.2–2.9	0.8

*(<37 weeks of gestation).

**Crude (Univariate).

***Adjusted (**Multivariate**).

### Determinants of HIV-free survival

**Figure 2:**
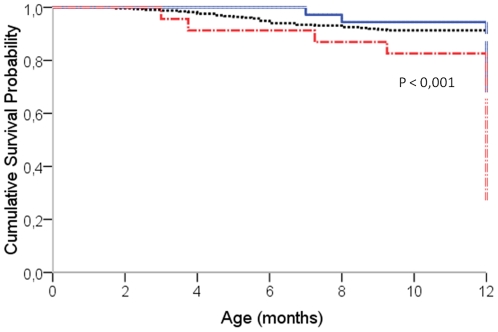
Cumulative Survival rates of children by 12 month according to mode of feeding, Cameroon. Taking in consideration defined feeding practice variables, survival curves by one year of age in each group were estimated using Kaplan Meier method. Next, survival curves between mixed-fed children (lower survival curve) and their counterpart exclusive formula-fed (middle survival curve) or exclusive breastfed (upper survival curve) were compared using the log-rank test.

The probability of progressing to infection or death at 12-month was 17% and the infant mortality rate was 9.1%. Taking into consideration feeding practices, infant mortality rate was 5.6% (95%CI: 0.9–17.2), 8.7% (95%CI: 6–12) and 21.7% (95%CI: 8.4–41.8), among children on EBF, EFF and MF respectively (p<0.001). By twelve month, infant mortality rates were 7.9% (95%CI: 5.5–11.1) and 20% (95%CI: 8.5–37) among uninfected and infected children respectively (P<0.001). Mixed-fed children had poorer survival outcomes by one year of age compare to their counterpart EBF or EFF (p<0.001) (figure 2). In univariate analysis, factors associated with an increased risk of being infected or dead were: duration of treatment shorter than 4 weeks [hazard ratio (HR): 3.6; 95%CI: 2.2–5.8], mothers on regimen 4 during pregnancy (HR: 4.2; 95%CI: 1.6–11), prematurity (HR = 4.3; 95%CI: 2.6–7.0), low birth weight (HR: 5.0; 95%CI: 3.1–8.1), EBF (HR = 2.8; 95%CI: 1.5–5.4) or mixed feeding (HR = 6.9; 95%CI: 3.9–12.4). In the multivariate analysis (table 2), prematurity, low birth weight and mixed feeding were still associated with the probability of progressing to infection or death.

**Table 2:** Determinants of risk of progression to HIV-infection or death (Multivariate Cox proportional Hazards model) at the CNPS hospital of Yaounde in Cameroon between 2004 and 2008.

	N	HR**	95% CI	P	HRa***	95% CI	*P*
**Duration of treatment**							
≥4 weeks before delivery	288	1			1		
<4 weeks before delivery	106	3.6	2.2–5.8	0.001	1.1	0.5–2.4	0.8
**Treatment**							
Regimen 1 (HAART)	66	1			1		
Regimen 2	153	1.8	0.7–4.7	0.3	0.9	0.3–2.8	0.9
Regimen 3	94	2.7	0.9–7.1	0.05	1.3	0.4–3.7	0.7
Regimen 4	81	4.2	1.6–11	0.004	1.9	0.6–6.5	0.3
**Feeding**							
Formula feeding	335	1			1		
Exclusive breastfeeding	36	2.8	1.5–5.4	0.02	2.1	0.9–6.0	0.06
Mixed Feeding	23	6.9	3.9–12.4	**0.0001**	8.7	3.6–20.6	**0.001**
**Birth weight**							
>2500 grams	305	1			1		
≤2500 grams	89	5.0	3.1–8.1	**0.001**	1.9	1.1–3.6	**0.03**
**Prematurity** *							
No	332	1			1		
Yes	62	4.3	2.6–7.0	**0.0001**	2.3	1.2–4.3	**0.009**

*(<37 weeks of gestation).

**Crude (Univariate).

***Adjusted (**Multivariate**).

## Discussion

Our study was based on a retrospective analysis of data from a routine PMTCT program in a reference hospital. To the best of our knowledge, this study presents primary results of the effectiveness of routine PMTCT interventions in Cameroon since the implementation of the new recommendations in 2004. Sources of data were patients' files and registers used for the monitoring of the services offered. Unfortunately, some pertinent variables for building a fully exploratory model such as mother's HIV-1 viral load [21], intermittent preventive treatment of malaria (IPT), child's vaccine status or prophylaxis with cotrimoxazole were not systematically recorded and could not be taken into account. The hospital where the study was carried out was reported to have good managerial and clinical practices. It was possible to access to register of the past four years as well as sources of information in antenatal, maternity, laboratory and paediatric services. All early HIV diagnosis and TCD4 lymphocytes counts were performed in the virology laboratory of the Centre Pasteur du Cameroun where results were stored in a computerized database. Few mother-infant pairs were excluded from the analysis (lost to follow-up or unexploitable files). Some of these infants might have been HIV-infected and this could have led to an overestimate of the effectiveness of the new regimens. Also, the association between duration of regimens and MTCT risk similarly could have been overestimated, since analysis were realised in an intent-to-treat basis. However, the observations we made in this reference health facility in Yaounde give reliable information of additional benefits gained by implementing multidrug ARV regimens in PMTCT. This might not be fully representative of results that would be expected from comparable PMTCT interventions in other sites in semi-urban or rural areas in Cameroon, where health workers may be less skilled, and operational contexts vary considerably.

In this study, the proportion of children with low birth weight was 22.3%. This proportion was greater than the 7.8% previously reported in Cameroon among children born to uninfected women [22], but was comparable to the figures reported in children born to HIV infected mothers having received similar ARV regimens for PMTCT in African settings with comparable HIV prevalence [11], [14]. The difference in weight observed between children born to HIV-positive women that were enrolled in this study and uninfected women in Cameroon could be due to their mothers' HIV serological status. The in utero exposure of children enrolled in this study to antiretroviral drugs could also be another explanation to this discrepancy [14], [23].

We observed three distinct feeding practices in our sample: exclusive formula feeding (86%), exclusive breastfeeding (9%) and mixed feeding (5%). Formula feeding of children born to HIV infected women had been previously described as acceptable and feasible in Yaounde [13]. The higher proportion of EFF observed in this study compared to other African population could be explained by a large number of mothers matching to AFASS (Acceptable, Feasible, Affordable, Sustainable, Safe) criteria according to WHO recommendations [24]. Formula feeding was reported feasible in relatively resource-limited settings if adequate water supply was available, and when women received intensive education and counselling [25]. Concerning mixed feeding which was found to be an important risk factor for infection or death, the proportion reported was as low as the 5% reported in a clinical research in Botswana [26]. A similar finding has been reported in Cameroon. A low proportion of mothers who switch to mixed feeding among a group of women who opted for formula feeding was observed. Such women rarely modify their feeding option by associating breastfeeding [27].

We observed programmatic changes in HAART eligibility during implementation of the public health program that we evaluated but this did not have an impact on MTCT rate in the two periods. Also, final results of children were considered regardless of the period since they were no difference in the sensitivity of the 2 methods as concerns detection of HIV infection at 6 weeks. The MTCT rate achieved with multidrug regimens at 6 weeks after birth was significantly lower than the 13.1% reported with HIVNET 012 protocol in Yaounde [13]. The result was close to the 7% reported in Botswana national PMTCT program [28]. It was also close to the 6.9% reported in a clinical trial in Malawi with a drug regimen equivalent to regimen 2 in our study [29]. Comparable results were also reported in the ANRS “Ditrame plus” study in Abidjan, where MTCT rates using protocols identical to regimen 2 and 3 were respectively 8.9% (95%CI: 5.3%–12.3%) and 5.9% (95%CI: 3.4%–8.5%) among children receiving FF. Duration of ARV regimens more than four weeks was very important for a better reduction of MTCT risk. Influence of a longer duration of antiretroviral prophylaxis on efficacy under research conditions has been reported earlier in Côte d'Ivoire [10] and in Botswana [30]. Altogether, sc ARV regimens in routine conditions in Cameroon showed a level of effectiveness comparable to the one observed during research activities [15] and can reduce the risk of MTCT to fewer than 7%. Therefore, our data show that regimens recommended by WHO in developing countries beyond sd-NVP, and outside of research settings, can be effective in routine use, although MTCT rate is still far from reaching the 5% target [31]. This should encourage key stakeholders and health professionals in limited settings to reinforce measures for increasing access to multidrug ARV regimens. Between 6 weeks and 12 months, the postpartum transmission due to breastfeeding was 25%. This was comparable to 14% and 29% reported in studies in developing countries [31], [32]. The probability of infection or death at 12-month was 17%. This did not vary significantly when comparing EBF and EFF groups. Infant mortality rates at 12-month were 7.5% and 20% respectively, among uninfected children and children found infected at 6 weeks. By age of one year, infant mortality among infected children was significantly higher. Early HIV-infection had been previously reported associated with a greater mortality among children [33]. In this study, the main risk factors of progressing to HIV-infection or death included mixed-feeding, pre-term birth and low-birth weight. These variables have also been reported as risk factors of infection or death in Abidjan [11].

In summary, this study showed that multidrug antiretroviral regimens recommended in 2004 were feasible and resulted in low MTCT rates under routine conditions. Poor obstetrical outcomes and mixed feeding appear as important operational risk factors which can erode the benefits of new regimens in routine use. This evaluation was made possible because early diagnosis of HIV-1 infection in children was available in Yaounde. However, it is worth noting that early diagnosis is not routinely available in health facilities nationwide. Hence, efforts made to effectively use dried blood spot (DBS), which have shown a great impact in the scaling-up of early diagnosis of HIV-1 need to be extended to the hinterlands where HIV diagnosis in children less than 9 months still remains inaccessible. Altogether, findings of this study could push decision-makers to enhance commitment and support an adequate and sustainable extension of the use of multidrug ARV regimens nationwide, within the PMTCT scaling-up, and to strengthen obstetrical care and infant nutrition in the context of HIV in low-income countries. This could also be an opportunity to improve mothers' access to antiretroviral prophylaxis and treatment and reduce HIV paediatric infections.

## References

[1] Tindyebwa D, Kayita J, Musoke P, Eley B, Nduati R (2006). Handbook on Paediatric AIDS in Africa.

[2] UNAIDS (2005). AIDS epidemic update: December 2005.

[3] UNAIDS (2007). AIDS epidemic update: December 2007.

[4] Mofenson LM, McIntyre JA (2000). Advances and research directions in the prevention of mother-to-child HIV-1 transmission.. Lancet.

[5] Coutsoudis A, Goga AE, Rollins N, Coovadia HM, on behalf of the child health Group (2002). Free formula milk for infants of HIV-infected women: blessing or curse?. Health Policy Plan.

[6] Nduati R, John G, Mbori-Ngacha D, Richardson B, Overbaugh J (2000). Effect of Breastfeeding and Formula feeding on Transmission of HIV-1: A randomized clinical trial.. JAMA.

[7] Stanton CK, Holtz SA (2006). Levels and trends in caesarean birth in the developing world.. Studies in Family Planning.

[8] Jackson JB, Musoke P, Fleming T, Guay LA, Bagenda D (2003). Intrapartum and neonatal single-dose nevirapine compared with zidovudine for prevention of mother-to-child transmission of HIV-1 in Kampala, Uganda: 18-month follow-up of the HIVNET 012 randomised trial.. Lancet.

[9] Guay LA, Musoke P, Fleming T, Bagenda D, Allen M (1999). Intrapartum and neonatal single-dose nevirapine compared with zidovudine for prevention of mother-to-child transmission of HIV-1 in Kampala, Uganda: HIVNET 012 randomised trial.. Lancet.

[10] Leroy V, Sakarovitch C, Cortina-Borja M, McIntyre J, Coovadia H (2005). Is there a difference in the efficacy of peripartum antiretroviral regimens in reducing mother-to-child transmission of HIV in Africa?. AIDS.

[11] Leroy V, Ekouevi DK, Becquet R, Viho I, Dequae-Merchadou L (2008). 18-Month Effectiveness of Short-Course Antiretroviral Regimens Combined with Alternatives to Breastfeeding to Prevent HIV Mother-to-Child Transmission.. PLoS ONE.

[12] WHO (2004). Antiretroviral drugs for treating pregnant women and preventing HIV infection in infants: guidelines on care, treatment and support for women living with HIV/AIDS and their children in resource-constrained settings.

[13] Tejiokem M, Nerrienet E, Tene G, Menu E, Barre-Sinoussi F (2004). Prevention of mother to child HIV-1 transmission (MTC) in Cameroon.. Med Mal Infect.

[14] Tonwe-Gold B, Ekouevi DK, Viho I, Amani-Bosse C, Toure S (2007). Antiretroviral treatment and prevention of peripartum and postnatal HIV transmission in West Africa: Evaluation of a two-tiered approach.. PLoS Med.

[15] Dabis F, Bequet L, Ekouevi DK, Viho I, Rouet F (2005). Field efficacy of zidovudine, lamivudine and single-dose nevirapine to prevent peripartum HIV transmission.. AIDS.

[16] Institut National de la Statistique Ministere de la Planification, de la Programmation du Developpement et de l'Amenagement du Territoire Yaounde (INS) [Cameroun] et ORC Macro International Inc (2004). Enquête Démographique et de Santé, Cameroun 2004.

[17] Mandelbrot L, Msellati P, Meda N, Leroy V, Likikouët R (2002). 15 Month follow up of African children following vaginal cleaning with benzalkonium chloride of their HIV infected mothers during late pregnancy and delivery.. Sex Transm infect.

[18] Alioum A, Cortina-Borja M, Dabis F, Dequae-Merchadou L, Haverkamp G (2003). Estimating the efficacy of interventions to prevent mother-to-child transmission of human immunodeficiency virus in breastfeeding populations: comparing statistical methods.. Am J Epidemiol.

[19] Armitage P, Berry G, Matthews JNS (2002). Statistical Methods in Medical Research. 4^th^ ed.

[20] Armitage P, Berry G, Matthews JNS (2002). Statistical Methods in Medical Research. 4^th^ ed.

[21] Jackson DJ, Chopra M, Doherty TM, Colvin MSE, Levin JB (2007). Operational effectiveness and 36 week HIV-free survival in the South African programme to prevent mother-to-child transmission of HIV-1.. AIDS.

[22] Piechulek H, Mendoza Aldana J (1996). Children with Low birth weight: The demands of a nutritional surveillance program, example: the rural zone of the littoral region (Cameroon).. Medecine d'Afrique Noire.

[23] Ekouevi D, Coffie P, Becquet R, Tonwe-Gold B, Horo A (2008). Antiretroviral therapy in pregnant women with advanced HIV disease and pregnancy outcomes in Abidjan, Cote d'Ivoire.. AIDS.

[24] WHO (2000). New Data on the Prevention of Mother-to-Child Transmission of HIV and their Policy Implications: Conclusions and Recommendations.

[25] Becquet R, Ekouevi DK, Viho I, Sakarovitch C, Toure H (2005). Acceptability of Exclusive Breast-Feeding With Early Cessation to Prevent HIV Transmission Through Breast Milk, ANRS 1201/1202 Ditrame Plus, Abidjan, Côte d'Ivoire.. J Acquir Immune Defic Syndr.

[26] Thior I, Lockman S, Smeaton LM, Shapiro RL, Wester C (2006). Breastfeeding Plus Infant Zidovudine Prophylaxis for 6 Months vs Formula Feeding Plus Infant Zidovudine for 1 Month to Reduce Mother-to-Child HIV Transmission in Botswana. A Randomized Trial: The Mashi Study.. JAMA.

[27] Njom Nlend A, Penda I, Same Ekobo C, Tene G, Tsague L (2007). Is exclusive artificial feeding feasible at 6 months post partum in Cameroon urban areas for HIV -Exposed infants?. J Trop Pediatr.

[28] Creek T, Tanuri A, Smith M, Seipone K, Smit M (2008). Early Diagnosis of Human Immunodeficiency Virus in Infants Using Polymerase Chain Reaction on Dried Blood Spots in Botswana's National Program for Prevention of Mother-to-Child Transmission.. Pediatr Infect Dis J.

[29] Taha ET, Kumwenda IN, Hoover RD, Fiscus AS, Kafulafula G (2004). Nevirapine and Zidovudine at Birth to Reduce Perinatal Transmission of HIV in an African Setting: A Randomized Controlled Trial.. JAMA.

[30] Shapiro RL, Thior I, Gilbert PB, Lockman S, Wester C (2006). Maternal single-dose nevirapine versus placebo as part of an antiretroviral strategy to prevent mother-to-child HIV transmission in Botswana.. AIDS.

[31] De Cock KM, Fowler MG, Mercier E, De Vincenzi I, Saba J (2000). Prevention of mother-to-child HIV transmission in resource-poor countries: translating research into policy and practice.. JAMA.

[32] Coutsoudis A, Pillay K, Kuhn L, Spooner E, Tsai WY (2001). Method of feeding and transmission of HIV-1 from mothers to children by 15 months of age: prospective cohort study from Durban, South Africa.. AIDS.

[33] Newell ML (2001). Prevention of mother-to-child transmission of HIV: challenges for the current decade.. Bull World Health Organ.

